# Purification of modified vaccinia virus Ankara from suspension cell culture

**DOI:** 10.1186/1753-6561-9-S9-O13

**Published:** 2015-12-14

**Authors:** Ingo Jordan, Diana Weimer, Stefan Iarusso, Holger Bernhardt, Verena Lohr, Volker Sandig

**Affiliations:** 1 ProBioGen AG, Goethestr. 54, 13086 Berlin, Germany; 2 Now at Sanofi-Aventis Deutschland GmbH, Industriepark Höchst, 65926 Frankfurt, Germany

## Background

A spectrum of viral vaccines, including the annual 620 million doses of trivalent influenza vaccines, are being produced in embryonated eggs of pharmaceutical quality, or with primary cells derived from such eggs [1,2]. Regulatory guidelines and experience for these processes are established and proven against time for 75 years [1]. However, production with galline primary material is not optimal and producers sometimes struggle to provide needed vaccine doses. Among the challenges are limitations in supply [3] and that rigid intervalls between husbandry, harvest of eggs and inoculation with vaccine seed must be accomodated [4]. Manipulation of embryonated eggs and disposal of solid biohazardous waste that accumulates if vaccines are being produced in egg cavities come at considerable costs [5,6]. Finally, risk of contamination with environmental and endogenous agents is high [7-9].

Such issues can be circumvented if a continuous cell line is used to propagate viral vaccines [1]. Master Cell Banks can be prepared in sufficient amounts and tested for presence of adventitious agents ahead of production, chemically defined media obviate dependence on animal derived components, and predictable seed trains towards a wide range of bioreactor volumes allow flexible and fast response times for vaccine production [10]. However, there is a regulatory concern that DNA derived from the immortal production substrate may be transferred to vaccine recipients [1]. Risk calculations that relate values for the length of typical oncogenes, number of such genes in the genome and fragmentation of DNA during purification have arrived at a permissive threshold of 10 ng of nucleic acid per vaccine dose [1].

## Materials and methods

We investigated purification of modified vaccinia virus Ankara (MVA) produced on the continuous avian cell line CR.pIX. MVA is a versatile and highly immunogenic viral vector, but also known to pose unique challenges in production processes [11]. For example, the majority of the infectious units of MVA remain cell-associated so that downstream purification must initiate with a complete lysate of the infected cultures (rather than cell-free supernatant). The viral particles are furthermore too large for conventional filtration, centrifugation and chromatographic separation. Finally, because MVA cannot amplify in human recipients, a desired safety feature, each vaccine dose requires 108 infectious units for full efficacy. This dose is 400-fold above that recommended for replication-competent poxviruses and necessitates efficient and robust manufacturing processes [10].

Derivation and properties of the anatine CR.pIX cell line have been described previously [12], as well as cell-associated propagation of poxviruses in suspension cultures in chemically-defined media by induction of CR.pIX aggregates [10]. We used GFP-recombinant versions of both genetically stable strains of MVA, wildtype and strain MVA-CR that was isolated previously with help of the suspension process [13].

Purification of MVA was performed with CIM monolithic chromatography columns from Bia Separations [14]. The CIM monoliths consist of a single piece of highly porous material with a network of branched channels. These channels can be provided with large 6 µm-diameters and can be functionalized with various ion exchange groups.

## Results

**Figure 1:**
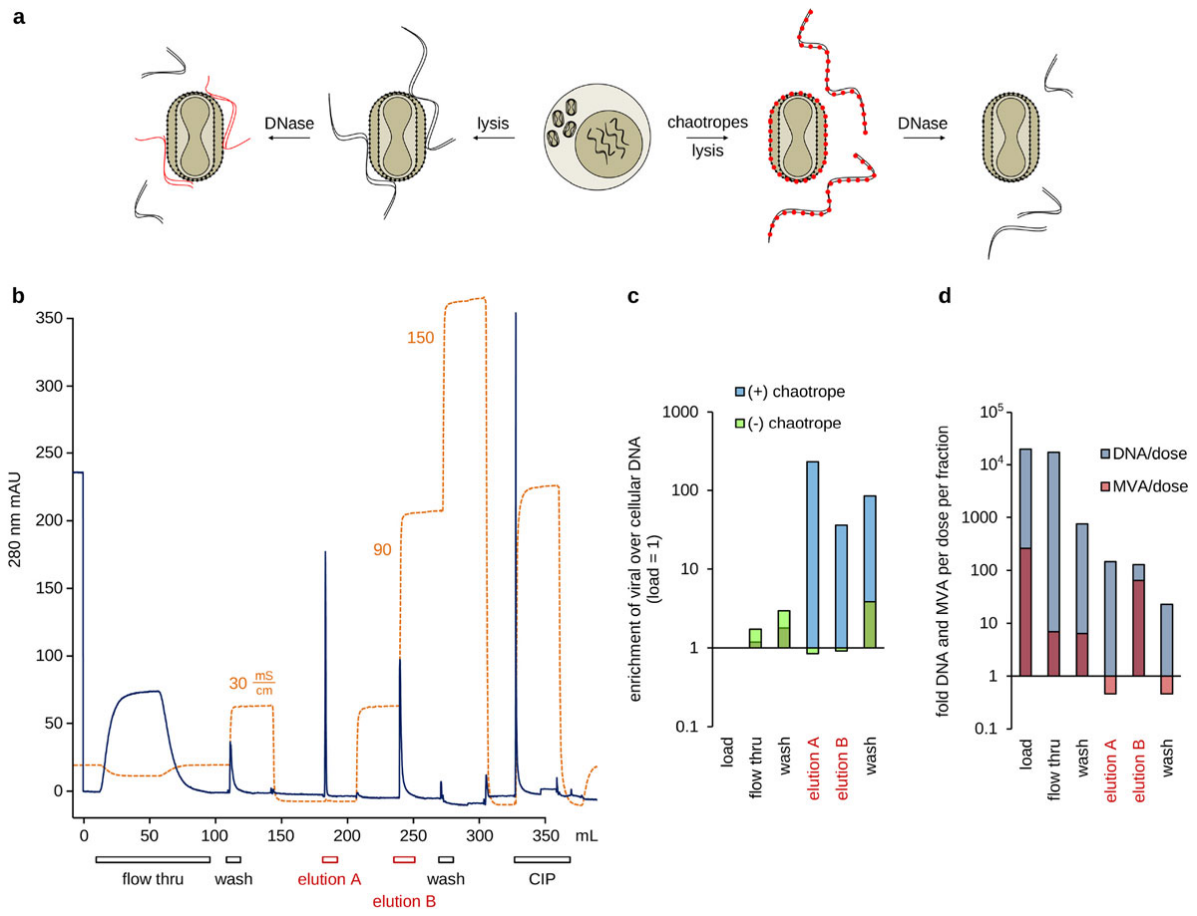
**Prevention of copurification of host-cell derived DNA**. (a) Enveloped virions appear to associate stably with host cell DNA. Chaotropes were added prior to lysis to prevent complex formation and to allow DNase to access its substrate without sterical hindrance. (b) A representative chromatogram of a large pore CIM ion exchange column loaded with lysate of MVA-infected CR.pIX cells. Elution fraction A is designed to remove DNA, the infectious units collect in B. (c) Quantification of the ratios of viral to cellular DNA by qPCR. The ratios of the load were set to 1. Blue columns show values obtained in presence and green in absence of chaotropes. (d) Quantification of yields relative to vaccine dose for a process with chaotropes.

Different monolith chemistries from cation and anion exchange to hydrophobic interaction were screened. However, host cell-derived DNA was difficult to deplete in these and other experiments. We suspected a strong association of viral envelopes and nucleic acids so that cellular DNA will always be copurified with the infectious units. Various chaotropic reagents were investigated to disrupt, preferrably to even prevent formation of these complexes [15]. Prevention of complex formation is an option because integrity of CR.pIX cells is maintained during MVA replication. The time for release of nuclear DNA in presence or absence of chaotropes during manufacturing can therefore be determined by the timing of sonication of the infected cultures (Figure 1 (a)).

A representative chromatogram obtained with a column at 1 mL scale is shown in Figure 1 (b). The load was adjusted to a conductivity of less than 10 mS/cm prior to application on the column. Washing was performed with 100 mM NaCl, elution by increasing the concentration of NaCl to conductivities of up to 150 mS/cm. Quantification of relative changes in DNA levels [16] by qPCR against the viral genome and an abundant cellular pseudogene demonstrated a strong enrichment of viral DNA only in presence of chaotropes (Figure 1 (c)). Without chaotropes the ratio of viral to cellular DNA remains close to that of the load. In subsequent experiments we measured an excess of 65 × for MVA (108 infectious units is 1-fold vaccine dose) and 130 × for the admissable DNA load (1-fold corresponds to 10 ng) in the product fraction (Figure 1 (d)).

## Conclusions

To replace eggs with a continuous avian cell line as vaccine production substrate is desirable but requires reduction of host cell DNA levels to less than 10 ng per vaccine dose. We used chaotropes to interfere with the association of cellular DNA and infectious units. This step, together with chromatography, yields a product fraction that is only 2-fold away from the admissable DNA load per efficaceous infectious activity. Next optimization steps will focus on improved processing of the lysate for additional depletion of cellular DNA.
